# Dietary inflammatory index and risk of upper aerodigestive tract cancer in Japanese adults

**DOI:** 10.18632/oncotarget.25288

**Published:** 2018-05-08

**Authors:** Makiko Abe, Nitin Shivappa, Hidemi Ito, Isao Oze, Tetsuya Abe, Yasuhiro Shimizu, Yasuhisa Hasegawa, Chikako Kiyohara, Masatoshi Nomura, Yoshihiro Ogawa, James R. Hebert, Keitaro Matsuo

**Affiliations:** ^1^ Department of Preventive Medicine, Kyushu University Faculty of Medical Sciences, Fukuoka, 812-8582, Japan; ^2^ Department of Medicine and Bioregulatory Science, Graduate School of Medical Science, Kyushu University, Fukuoka, 812-8582, Japan; ^3^ Division of Molecular and Clinical Epidemiology, Aichi Cancer Center Research Institute, Nagoya, 464-8681, Japan; ^4^ Cancer Prevention and Control Program, University of South Carolina, Columbia, SC, 29208, USA; ^5^ Department of Epidemiology and Biostatistics, Arnold School of Public Health, University of South Carolina, Columbia, SC, 29208, USA; ^6^ Connecting Health Innovations LLC, Columbia, SC, 29201, USA; ^7^ Department of Epidemiology, Nagoya University Graduate School of Medicine, Nagoya, 466-8550, Japan; ^8^ Department of Gastrointestinal Surgery, Aichi Cancer Center Hospital, Nagoya, 464-8681, Japan; ^9^ Department of Head and Neck Surgery, Aichi Cancer Center Hospital, Nagoya, 464-8681, Japan; ^10^ Department of Endocrinology and Metabolism, Kurume University School of Medicine, Kurume, 830-0011, Japan; ^11^ Department of Molecular and Cellular Metabolism, Graduate School of Medical and Dental Sciences, Tokyo Medical and Dental University, Tokyo, 113-8510, Japan

**Keywords:** upper aerodigestive tract cancer, dietary inflammatory index, Japanese adults, persistent infection, case-control study

## Abstract

**Background:**

The inflammatory potential of diet that has been shown to be associated with cancer risk. We examined the association between dietary inflammatory potential as measured by the dietary inflammatory index (DII^®^) and risk of upper aerodigestive tract cancers in a Japanese case-control study.

**Results:**

A positive association was observed between increasing DII scores and overall upper aerodigestive tract cancers, and across anatomic subsites. For upper aerodigestive tract cancers, the OR_Q4vsQ1_ = 1.73 (95% CI: 1.37–2.20); head and neck cancer, the OR_Q4vsQ1_ was 1.92 (95% CI: 1.42–2.59); and for esophageal cancer, the OR_Q4vsQ1_ was1.71 (95% CI: 1.54–1.90). Risks for hypopharyngeal and nasopharyngeal cancers were greatly elevated: (OR_Q4vsQ1_ = 4.05 (95% CI: 1.24–13.25) for hypopharyngeal cancer and OR_Q4vsQ1_ = 4.99 (95% CI: 1.14–21.79) for nasopharyngeal cancer.

**Conclusion:**

A more pro-inflammatory diet was associated with an elevated risk of upper aerodigestive tract cancers after accounting for important confounders. All anatomic subsites, except larynx, showed the consistently elevated risk with increasing DII score. Those subsites with known etiological associations with persistent infection showed the largest elevation in risk. These results warrant further evaluation in future studies.

**Materials and Methods:**

This is a case-control study of 1,028 cases and 3,081 age- and sex-matched non-cancer controls recruited at Aichi Cancer Center. DII scores were computed based on estimates of macro- and micro-nutrients from a self-administered food frequency questionnaire. Scores were further categorized into quartiles (based on the distribution in controls). Conditional logistic regression models were fit to estimate odds ratio (OR) and 95% confidence intervals (CIs) adjusted for smoking, ethanol consumption, alcohol flushing, number of teeth, and occupation group.

## INTRODUCTION

Upper aerodigestive tract cancers (UATC), encompassing the oral cavity, pharynx, larynx, salivary glands and esophagus, represent a significant cancer burden; collectively ranking fourth for cancer incidence and second for cancer mortality worldwide [1]. Smoking and excessive alcohol drinking are the two prominent risk factors for UATC [2–6]. In addition, a probable role of dietary exposure has been reported [7–9]; with non-starchy vegetables, and fruits having a protective role, and excess drinking, intake of red meat, and processed meat having a carcinogenic role [10]. Current evidence also indicates that diet plays a role in regulating inflammatory processes [11] by modulating the levels of inflammatory cytokines such as c-reactive protein (CRP), interleukin (IL)-6 and tumor necrosis factor (TNF)-α [12–14]. These components are associated with insulin resistance, adiposity, metabolic syndrome, and cardiovascular disease, and they have been shown to increase UATC risk by promoting proliferation, angiogenesis, and other mechanisms of carcinogenesis [15–24].

The literature-derived dietary inflammatory index (DII^®^) was developed to measure the inflammatory potential of diet in relation to six inflammatory markers [25], including CRP [14], and IL-6 [26]. The DII has been associated with various neoplasms including esophageal [27], laryngeal [28], pharyngeal [29], colorectal [30], prostate [31], pancreatic [32], endometrial [33], and hepatocellular cancers [34]. Most DII research has been conducted primarily in European and European-American populations [35, 36]. In contrast, among Asians, including Japanese, epidemiologic evidence about the association between inflammatory potential of diet and cancer risk is sparse. Therefore, we investigated the association between inflammatory potential of dietary intake and UATC risk with in relation to DII scores among Japanese adults.

Our objective in this study was to examine whether more pro-inflammatory diets, as measured by DII, are associated with a higher risk of UATC and if there is an interaction between DII scores and potential effect modifiers.

## RESULTS

Table 1 presents baseline characteristics of the cases and controls. Smoking, alcohol consumption, flushing phenotype, number of teeth, and occupation group were statistically significantly different between cases and controls in all UATC analysis. These differences also were observed in both head and neck cancer and esophagus cancers when analyzed separately. Smoking and alcohol consumption were more prevalent among cases compared with controls, and there were more blue-collar workers than white collar workers among cases. Absence of flushing after drinking and fewer teeth were more prevalent in cases. In further analyses, these variables were considered confounders.

**Table 1:** Baseline characteristics of study subjects

	Head and neck + Esophagus				Head and neck				Esophagus		
	Case (%)	Control (%)	*P* value		Case (%)	Control (%)	*P* value		Case (%)	Control (%)	*P* value
**All male + female)**	1028	3081	0.83	**All (male + female)**	595	1,785	0.87	**All (male + female)**	433	1,296	0.89
male	826 (80.4)	2,466 (80.0)		male	450 (75.6)	1,344 (75.3)		male	376 (86.8)	1,122 (86.6)	
female	202 (19.7)	615 (20.0)		female	145 (24.4)	441 (24.7)		female	57 (13.2)	174 (13.4)	
**Age (mean, SD)**	(60, 10.9)	(60, 10.8)	0.73	**Age (mean, SD)**	(60, 10.9)	(60, 10.8)	0.99	**Age (mean, SD)**	(60,10.9)	(60, 10.8)	0.62
<40	56 (5.5)	171 (5.6)		<40	53 (8.9)	164 (9.2)		<40	3 (0.7)	7 (0.5)	
40-49	89 (8.7)	276 (9.0)		40-49	69 (11.6)	209 (11.7)		40-49	20 (4.6)	67 (5.2)	
50-59	306 (29.8)	907 (29.4)		50-59	173 (29.1)	514 (28.8)		50-59	133 (30.7)	393 (30.3)	
60-69	364 (35.4)	1,144 (37.1)		60-69	184 (30.9)	565 (31.7)		60-69	180 (41.6)	579 (44.7)	
≥70	213 (20.7)	583 (18.9)		≥70	116 (19.5)	333 (18.7)		≥70	97 (22.4)	250 (19.3)	
**Smoking**			<0.001	**Smoking**			<0.001	**Smoking**			<0.001
Non	201 (19.6)	1,135 (36.8)		Non	152 (25.6)	697 (39.1)		Non	49 (11.3)	438 (33.8)	
Low-Modarate	122 (11.9)	548 (17.8)		Low-Modarate	84 (14.1)	345 (19.3)		Low-Modarate	38 (8.8)	203 (15.7)	
High-Moderate	276 (26.9)	636 (20.6)		High-Moderate	144 (24.2)	344 (19.3)		High-Moderate	132 (30.5)	292 (22.5)	
Heavy	413 (40.2)	730 (23.7)		Heavy	204 (34.3)	385 (21.6)		Heavy	209 (48.3)	345 (26.6)	
Unknown	16 (1.6)	32 (1.0)		Unknown	11 (1.9)	14 (0.8)		Unknown	5 (1.2)	18 (1.4)	
**Alcohol consumption**			<0.001	**Alcohol consumption**			<0.001	**Alcohol consumption**			
Non	199 (19.4)	1,019 (33.1)		Non	159 (26.7)	638 (35.7)		Non	40 (9.2)	381 (29.4)	
Moderate	176 (17.1)	848 (27.5)		Moderate	121 (20.3)	475 (26.6)		Moderate	55 (12.7)	373 (28.8)	
1-2 go×5/week	229 (22.3)	711 (23.1)		1-2 go×5/week	123 (20.7)	392 (22.0)		1-2 go×5/week	106 (24.5)	319 (24.6)	
>1-2 go×5/week	401 (39.0)	467 (15.2)		>1-2 go×5/week	175 (29.4)	264 (14.8)		>1-2 go×5/week	226 (52.2)	203 (15.7)	
Unknown	23 (2.2)	36 (1.2)		Unknown	17 (2.9)	16 (0.9)		Unknown	6 (1.4)	20 (1.5)	
**Flushing phenotype**			<0.001	**Flushing phenotype**			<0.001	**Flushing phenotype**			
Yes	443 (43.1)	1,555 (59.5)		Yes	263 (44.2)	900 (50.4)		Yes	180 (41.6)	655 (50.5)	
No	544 (52.9)	1,455 (47.2)		No	302 (50.8)	843 (47.2)		No	242 (55.9)	612 (47.2)	
Unknown	41 (4.0)	71 (2.3)		Unknown	30 (5.0)	42 (2.4)		Unknown	11 (2.5)	29 (2.2)	
Teeth			<0.001	Teeth			<0.001	Teeth			<0.001
0	79 (7.7)	121 (3.9)		0	43 (7.2)	78 (4.4)		0	79 (7.7)	121 (3.9)	
1-8	199 (19.4)	374 (12.1)		1-8	111 (18.7)	193 (10.8)		1-8	199 (19.4)	374 (12.1)	
9-20	309 (30.1)	879 (28.5)		9-20	168 (28.2)	499 (28.0)		9-20	309 (30.1)	879 (28.5)	
≥21	427 (41.5)	1,673 (54.3)		≥21	264 (44.4)	991 (55.5)		≥21	427 (41.5)	1,673 (54.3)	
Unknown	14 (1.4)	34 (1.1)		Unknown	9 (1.5)	24 (1.3)		Unknown	14 (1.4)	34 (1.1)	
**Occupation group**			<0.001	**Occupation group**			<0.001	**Occupation group**			<0.001
Blue collar	381 (37.1)	841 (27.3)		Blue collar	221 (37.1)	512 (28.7)		Blue collar	160 (37.0)	329 (25.4)	
White collar	217 (21.1)	917 (29.8)		White collar	131 (22.0)	529 (29.6)		White collar	86 (19.9)	388 (29.9)	
Other	417 (40.6)	1,272 (41.3)		Other	235 (39.5)	719 (40.3)		Other	182 (42.0)	553 (42.7)	
Unknown	13 (1.3)	51 (1.7)		Unknown	8 (1.3)	25 (1.4)		Unknown	5 (1.2)	26 (2.0)	
**Subsite**			1.00	Subsite				Subsite			
**Head and neck cancer**											
Oral cavity	255 (24.8)	762 (24.7)									
Nasopharynx	50 (4.9)	153 (5.0)									
Oropharynx	72 (7.0)	214 (7.0)									
Hypopharynx	80 (7.8)	240 (7.8)									
Larynx	92 (9.0)	275 (8.9)									
Salivary gland	23 (2.2)	72 (2.3)									
Other HNC	23 (2.2)	69 (2.2)									
**Esophagus**	433 (42.1)	1,296 (42.1)									

Table 2 presents the distribution of characteristics across quartiles of DII. DII scores, which ranged from –4.31 (most anti-inflammatory) to +2.02 (most pro-inflammatory). We observed a statistically significant association between DII quartiles and smoking, alcohol and occupational group, but not flushing phenotype and number of teeth. Participants with higher DII scores tended to be male, younger, heavier smokers or drinkers, and blue-collar workers, compared to subjects with lower DII scores. Supplementary Table 2 presents the distribution of macro-and micro-nutrients and major food groups across DII quartiles among controls. All the nutrients showed statistically significant associations acorss DII quartiles. Carbohydrate showed a positive association with DII scores, while the others showed negative correlation. Food groups such as meat, fish, seafoods other than fish, green-yellow vegetables, other vegetables, fruits and soy also had statistically significant associations with DII quartiles and showed negative correlation with DII scores.

**Table 2:** DII distribution according to confounders among controls

Controls	DII quartiles (%)				
	1 (−4.31– −1.00)	2 (−1.00–0.11)	3 (0.11–0.58)	4 (0.58–2.02)	*P* value
**Sex**					<0.001
Male	538 (21.8)	607 (24.6)	628 (25.5)	693 (28.1)	
Female	233 (37.9)	163 (26.5)	142 (23.1)	77 (12.5)	
**Age**					<0.001
<40	30 (17.5)	34 (19.9)	37 (21.6)	70 (40.9)	
40–49	54 (19.6)	57 (20.7)	84 (30.4)	81 (29.4)	
50–59	180 (19.9)	221 (24.4)	262 (28.9)	244 (26.9)	
60–69	317 (27.7)	295 (25.8)	261 (22.8)	271 (23.7)	
70-	190 (32.6)	163 (28.0)	126 (21.6)	104 (17.8)	
**Smoking**					<0.001
Non	378 (33.3)	279 (24.6)	254 (22.4)	224 (19.7)	
Low-Modarate	114 (20.80)	141 (25.7)	147 (26.8)	146 (26.6)	
High-Moderate	137 (21.5)	169 (26.6)	163 (25.6)	167 (26.3)	
Heavy	140 (19.2)	172 (23.6)	197 (27.0)	221 (30.3)	
Unknown	2 (6.3)	9 (28.1)	9 (28.1)	12 (37.5)	
**Alcohol consumption**					0.02
Non	269 (26.4)	259 (25.4)	244 (24.0)	247 (24.2)	
Moderate	219 (25.8)	196 (23.1)	207 (24.4)	226 (26.7)	
1-2 go×5/week	191 (26.9)	185 (26.0)	171 (24.1)	164 (23.1)	
>2 go×5/week	83 (17.8)	119 (25.5)	137 (29.3)	128 (27.4)	
Unknown	9 (25.0)	11 (30.6)	11 (30.6)	5 (13.9)	
**Flushing phenotype**					0.19
Yes	383 (24.6)	383(24.6)	377 (24.2)	412 (26.5)	
No	363 (25.0)	368 (25.3)	378 (26.0)	346 (23.8)	
Unknown	25 (35.2)	19 (26.8)	15 (21.1)	12 (16.9)	
**Teeth**					0.62
0	28 (23.1)	33 (27.3)	30 (24.8)	30 (24.8)	
1-8	93 (24.9)	98 (26.2)	81 (21.7)	102 (27.3)	
9-20	218 (24.8)	208 (23.7)	231 (26.3)	222 (25.3)	
≥21	424 (25.3)	427 (25.5)	419 (25.0)	403 (24.1)	
Unknown	8 (23.5)	4 (11.8)	9 (26.5)	13 (38.2)	
**Occupation group**					<0.001
Blue collar	143 (17.0)	191 (22.7)	230 (27.4)	277 (32.9)	
White collar	198 (21.6)	241 (26.3)	234 (25.5)	244 (26.6)	
Other	411 (32.3)	323 (25.4)	295 (23.2)	243 (19.1)	
Unknown	19 (37.3)	15 (29.4)	11 (21.6)	6 (11.8)	

Table 3 shows the association between DII scores and risk for all UATC, and separately for head and neck, and esophageal cancer. A more pro-inflammatory diet was associated with an increased risk of UATC (OR_Q4vsQ1_: 1.96; 95% CI: 1.58–2.43; *P*-trend < 0.001). This finding was essentially unchanged after adjusting for potential confounders (OR_Q4vsQ1_: 1.73, 1.37–2.20; *P*-trend < 0.001). Similarly, an inflammatory diet was associated with increased risk of head and neck cancer even after adjustment of confounders (OR_Q4vsQ1_: 1.92; 1.42–2.59; *P*-trend < 0.001). Adjusted OR_Q4vsQ1_ remained significant for esophageal cancer (1.71; 1.54–1.90); however, a linear trend was attenuated compared with UATC overall (*P*-trend 0.07). Table 4 shows results according to subsite in head and neck cancer. Among these, all but laryngeal cancers produced results consistent with overall UATC. Notably, nasopharyngeal cancer and hypopharyngeal cancer showed strong positive associations, even in second and third quartiles, compared with the lowest quartile.

**Table 3:** Impact of DII and selected variables

	DII quartiles	Case/control	OR (95% CI)^a^	Adjusted OR (95% CI)^b^
**Head and neck + Esophagus**				
	1 (−4.31 – −1.00)	174/771	1.00 (Reference)	1.00 (Reference)
	2 (−1.00 – −0.11)	243/770	1.41 (1.13–1.76)	1.33 (1.04–1.69)
	3 (0.11–0.58)	278/770	1.62 (1.31–2.01)	1.39 (1.10–1.76)
	4 (0.58–2.02)	333/770	1.96 (1.58–2.43)	1.73 (1.37–2.20)
	***P* for trend**		<0.001	<0.001
**Head and neck**				
	1 (−4.08 – −1.13)	96/443	1.00 (Reference)	1.00 (Reference)
	2 (−1.13 – −0.12)	129/447	1.35 (1.00–1.81)	1.33 (0.97–1.82)
	3 (−0.12 − 0.52)	168/449	1.76 (1.33–2.35)	1.57 (1.17–2.13)
	4 (0.52–1.99)	202/446	2.16 (1.63–2.87)	1.92 (1.42–2.59)
	***P* for trend**		<0.001	<0.001
**Esophagus**				
	1 (−4.31 – −1.03)	78/328	1.00 (Reference)	1.00 (Reference)
	2 (−1.03 – −0.11)	114/323	1.50 (1.08–2.10)	1.11 (0.99–1.25)
	3 (0.11–0.57)	110/321	1.45 (1.04–2.02)	1.42 (1.29–1.57)
	4 (0.57–2.02)	131/324	1.72 (1.24–2.38)	1.71 (1.54–1.90)
	***P* for trend**		0.003	0.07

^a^Odds ratios were estimated by conditional logistic models not adjusted for any covariates.

^b^Odds ratios were estimated by conditional logistic models adjusted for smoking, ethanol consumption, flushing phenotype, teeth and occupation group.

**Table 4:** Imapct of DII according to detailed subsites in head and neck cancer

Head and neck				
Subsites	DII quartiles	Case/control	OR (95% CI)^a^	Adjusted OR (95% CI)^b^
**Oral cavity**	1 (−4.08 – −1.13)	47/196	1.00 (Reference)	1.00 (Reference)
	2 (−1.13 – −0.12)	50/183	1.17 (0.74–1.84)	1.21 (0.75–1.96)
	3 (−0.12–0.52)	62/205	1.32 (0.86–2.03)	1.25 (0.79–1.96)
	4 (0.52–1.99)	96/178	2.42 (1.58–3.71)	2.38 (1.52–3.72)
	***P* for trend**		<0.001	<0.001
**Nasopharynx**	1 (−4.08 – −1.13)	4/37	1.00 (Reference)	1.00 (Reference)
	2 (−1.13 – −0.12)	12/36	3.24 (0.92–11.46)	5.71 (1.05–30.89)
	3 (−0.12 – 0.52)	17/35	4.88 (1.47–16.17)	7.78 (1.65–36.57)
	4 (0.52 – 1.99)	17/45	3.65 (1.10–12.11)	4.99 (1.14–21.79)
	***P* for trend**		0.05	0.09
**Oropharynx**	1 (−4.08 – −1.13)	12/56	1.00 (Reference)	1.00 (Reference)
	2 (–1.13 – −0.12)	18/52	1.62 (0.69–3.80)	2.18 (0.82–5.83)
	3 (−0.12 – 0.52)	22/49	2.02 (0.92–4.42)	2.14 (0.87–5.24)
	4 (0.52 – 1.99)	20/57	1.63 (0.72–3.69)	1.71 (0.65–4.50)
	***P* for trend**		0.21	0.27
**Hypopharynx**	1 (−4.08 – −1.13)	6/51	1.00 (Reference)	1.00 (Reference)
	2 (−1.13 – −0.12)	23/61	3.29 (1.24–8.71)	4.39 (1.36–14.11)
	3 (−0.12 – 0.52)	29/65	4.18 (1.55–11.26)	4.59 (1.45–14.51)
	4 (0.52 – 1.99)	22/63	3.03 (1.15–7.97)	4.05 (1.24–13.25)
	***P* for trend**		0.07	0.04
**Larynx**	1 (−4.08 – −1.13)	18/56	1.00 (Reference)	1.00 (Reference)
	2 (−1.13 – −0.12)	15/78	0.63 (0.30–1.33)	0.39 (0.16–0.99)
	3 (−0.12 – 0.52)	30/62	1.53 (0.77–3.06)	1.02 (0.43–2.44)
	4 (0.52 – 1.99)	29/79	1.17 (0.59–2.33)	0.59 (0.25–1.38)
	***P* for trend**		0.21	0.68
**Salivary gland**	1 (−4.08 – −1.13)	5/23	1.00 (Reference)	1.00 (Reference)
	2 (−1.13 – −0.12)	6/21	1.35 (0.38–4.88)	1.57 (0.37–6.63)
	3 (−0.12 – 0.52)	4/15	1.41 (0.32–6.16)	1.07 (0.19–5.97)
	4 (0.52 – 1.99)	8/13	5.42 (1.01–29.17)	2.91 (0.40–21.19)
	***P* for trend**		0.08	0.13

^a^Odds ratios were estimated by conditional logistic models not adjusted for any covariates.

^b^Odds ratios were estimated by conditional logistic models adjusted for smoking, ethanol

consumption, flushing phenotype, teeth and occupation group.

Table 5 shows the adjusted ORs of UATC according to strata of selected confounders: age, sex, smoking, alcohol consumption, flushing phenotype, number of teeth, occupation group and their interactions. Although there is apparent variability in the impact of the association according to strata, positive associations between DII and UATC risk were consistently observed. We explored alternative analyses using the DII computed with alcohol included and confirmed that results were consistent with those based on the DII not including alcohol (data not shown).

**Table 5:** Results for stratified analysis by selected variables

Head and neck + Esophagus							
DII quartiles							
		1 (−4.31 – −1.00)	2 (–1.00 –0.11)	3 (0.11–0.58)	4 (0.58–2.02)		
Variables	Case/control (*n*) Multivariate OR^a^ (95% CI)					*P* for trend	interaction *P*^*b*^
**Sex**	Male	134/538	192/607	225/628	275/693	0.012	
		1.00 (Reference) 1.17 (0.88–1.55)	1.21 (0.92–1.59)	1.41 (1.08–1.85)			
	Female	40/233	51/163	53/142	58/77	<0.001	<0.001
		1.00 (Reference) 1.88 (1.13–3.13)	1.91 (1.15–3.15)	3.87 (2.22–6.75)			
**Age**	<60	61/264	89/312	113/383	188/395	0.001	
		1.00 (Reference) 1.07 (0.71–1.61)	1.16 (0.78–1.72)	1.78 (1.22–2.60)			
	≥60	113/507	154/458	165/387	145/375	0.006	0.18
		1.00 (Reference) 1.52 (1.11–2.08)	1.67 (1.22–2.28)	1.54 (1.12–2.14)			
**Smoking**	Never	45/378	48/279	45/254	63/224	<0.001	
		1.00 (Reference) 1.74 (0.98–3.08)	1.91 (1.08–3.36)	3.03 (1.65–5.55)			
	Ever	129/393	194/490	233/516	269/543	0.007	0.021
		1.00 (Reference)	1.22 (0.90–1.66)	1.37 (1.03–1.84)	1.48 (1.10–1.98)		
**Alcohol**	Never	37/26941/259	58/244	63/247	<0.001		
**consumption**		1.00 (Reference)	1.14 (0.61–2.13)	1.85 (1.02–3.34)	3.21 (1.68–6.14)		
	Ever	137/502	201/510	220/526	270/523	0.003	0.58
		1.00 (Reference)	1.25 (0.93–1.68)	1.17 (0.88–1.56)	1.59 (1.20–2.12)		
**Flushing**	Yes	79/383	97/383	124/377	143/412	0.040	
**phenotype**		1.00 (Reference)	1.09 (0.69–1.72)	1.56 (1.01–2.42)	1.46 (0.93–2.30)		
	No	88/363	137/368	140/378	179/346	0.005	0.67
		1.00 (Reference)	1.49 (0.96–2.32)	1.05 (0.67–1.64)	2.05 (1.34–3.13)		
**Teeth**	0–8	48/121	77/131	65/111	88/132	0.540	
		1.00 (Reference)	2.11 (0.88–5.05)	1.40 (0.57–3.44)	1.62 (0.62–4.25)		
	9–20	58/218	68/208	89/231	94/222	0.460	0.49
		1.00 (Reference)	0.87 (0.45–1.68)	1.00 (0.54–1.88)	1.22 (0.65–2.29)		
	≥21	68/424	96/427	118/419	145/403	0.002	
		1.00 (Reference)	1.20 (0.77–1.88)	1.44 (0.94–2.20)	1.86 (1.22–2.85)		
**Occupation**	Blue collar	56/143	89/191	99/230	137/277	0.410	
**group**		1.00 (Reference)	0.79 (0.39–1.59)	0.80 (0.41–1.54)	1.18 (0.61–2.26)		
	White collar	38/198	40/241	62/234	77/244	0.080	<0.001
		1.00 (Reference)	0.91 (0.39–2.13)	1.15 (0.54–2.48)	1.78 (0.82–3.86)		
	Other	78/411	112/323	113/295	114/243	0.005	
		1.00 (Reference)	1.70 (1.11–2.60)	1.61 (1.06–2.46)	1.97 (1.27–3.08)		

^a^Odds ratios were estimated by conditional logistic models adjusted for smoking, ethanol consumption, flushing, phenotype, teeth and occupation group.

^b^Interactions were evaluated by likelihood ratio test between a model including a cross-product term between variables of interest and DII quartiles and a model without the term.

## DISCUSSION

For the first time in a Japanese population, this case-control study examined the association between inflammatory potential of diet, as estimated by the DII, and risk of UATC for the first time in a Japanese population. We found consistently significant positive associations with UATC risk even after accounting for potential confounders. Results were consistent among head and neck cancer and esophageal cancer. We also found that the strength of the association with DII scores was heterogeneous across subsites in head and neck cancer: nasopharyngeal and hypopharyngeal cancer showed increased risk at even in DII quartiles 2 and 3. In contrast, laryngeal cancer showed no obvious association with DII.

Our overall findings are in accordance with previous reports showing that a pro-inflammatory diet, as indicated by higher DII scores, was associated with UATC risk [27–29, 42–44]. Our findings also are consistent with previous results showing that smoking and alcohol drinking are established risk factors for UATC and our finding is in consistent with former findings. Constituents in cigarette smoke constituents, particularly reactive oxidative substances (ROS), activate epithelial cell intracellular signaling cascades that lead to inflammatory gene activation (e.g., IL-8 and TNF-α), and the secretion of those inflammatory mediators promotes chronic immune cell recruitment and inflammation [45]. Similarly, chronic ethanol exposure also induces inflammation; ethanol toxicity is associated with the induction of NF-κB that results in the expression of inflammatory mediators including cytokines (TNF-α, IL-6 and IL-12), lipid mediators, inducible nitric oxide synthase (iNOS) and cyclooxigenase-2 (COX-2) [46]. Therefore, a significantly positive association between DII and UATC risk seems biologically plausible.

Interestingly, we observed marked impact of even minor increases in DII scores in increasing hypopharyngeal and nasopharyngeal cancer risk. Both sites are known to be associated with infections by human papilloma virus (HPV) [47–50] and Epstein-Barr virus (EBV) [51, 52], respectively. Inflammation caused by chronic infection is regarded as a major risk factors for various types of cancer, and underlying infections and inflammation are linked to 15–20% of all cancer deaths [53]. Immune response to pathogens that establish persistent infections is designed to promote host defense; however, it also can stimulate chronic inflammation and tumor growth [54]. HPV infection produces and releases several inflammatory cytokines from keratinocytes, their main target cell type; from skin fibroblasts; and form different components of the innate and adaptive immune response, including macrophages, natural killer cells and lymphocytes. On the other hand, infection of EBV results in the activation of STAT3 and NF-κB signal cascades in target epithelial cells, which induces increased expression of inflammatory cytokines including IL-6 and COX-2 [55]. Persistent infections with these viruses have been linked to chronic inflammation, an important factor for cancer development [56]. Taking these facts into consideration, our finding suggests that the pro-inflammatory potential of diet, as indicated by higher DII scores, significantly increases risk of infection-related cancers.

Previous reports revealed protective effect of vegetable, fruits [57, 58], olive oil [59], fish [29, 60], whole grains [61, 62], vitamin [63, 64], folate [65, 66] and fiber [63, 67]; whereas there appears to be a carcinogenic effect of red and processed meat [68, 69], fat [58, 70] and carbohydrate [71, 72] for UATC. These foods and nutrients, all components of DII, have the potential to induce the inflammatory response by influencing inflammatory markers such as CRP, IL-1β, IL-4, IL-6, IL-10 and TNF-α [12, 13, 73]. Particularly, CRP, IL-6 and TNF-α, in particular, have been reported to be associated with a variety of cancers [74–78]. Cyclooxygenase pathway products with the potential to influence inflammation include reactive oxygen species (ROS) and nitric oxide (NO). These can damage DNA and other cellular macromolecules. This damage results in increased proliferation, mutations, DNA damage and angiogenesis [75, 79, 80]. Among inflammatory markers, IL-1βhas been demonstrated to induce the production of gelatinases, which are family members of the matrix metalloproteinases (MMPs) that contribute to tumor invasion and metastasis [81], whereas IL-4 and IL-10 play a role in immune suppression, and IL-6 plays a role in anti-apoptosis [74] for head and neck cancer.

The methodological strengths of this study include its large sample size with very high (96.7%) response rate. Second, the FFQ that provided the data for DII estimation was tested for validity and reproducibility [39, 82]. Third, potential confounding by age, sex and other factors was addressed by matching and statistical adjustment.

Despite its strengths, potential limitations of our study also warrant mention. First, we collected information about confounders via self-reported questionnaire, therefore, it is difficult to rule out potential sources of information bias. If present, however, the effect of such misclassification in relation to possible under-adjustment would be limited. Also consistency of results across stratified analysis by several potential confounders is reassuring. Second, the control participants were selected among non-cancer patients at our hospital. Because cases and controls were selected from the same hospital and almost all patients lived in the Tokai area of central Japan, the internal validity of this case–control study is likely to be acceptable [83].

Third, by not setting eligibility criteria for control diseases it is possible that certain specific diagnostic groups may be related exposures of interest. Fourth, the limited number of cases for particular subsites reduces statistical power. Fifth, because of the retrospective nature of data collection, information bias in responses to the FFQ cannot be ruled out. Sixth, no validation study on the DII has been conducted in Japan. However, the DII was designed for universal applicability and has been construct validated in numerous populations including Asian countries such as Korea [84] and Iran [85]. All of these validation studies produced essentially identical results. Seventh, 22 food parameters were not-available to calculate DII scores. Previously, we showed that DII scores calculated from fewer than 22 food parameters were associated with inflammation [86]. Some of the food parameters that are missing include ginger, turmeric, saffron, thyme, eugenol – all of which are not consumed in high amounts in this population. However, presence of some missing parameters, such as various flavonoids, which are consumed regularly, could have influenced the results.

In conclusion, we found a positive association between intake of a pro-inflammatory diet, as indicated by high DII score, and risk of UATC in Japanese adults. Results persisted after adjusting for potential confounders, including smoking and drinking. The association was consistently observed in esophageal cancer and most of UATC subsites, except laryngeal cancer. The fact that subsites which are known to have an etiological association with persistent infection; i.e., nasopharyngeal and hypopharyngeal cancers, showed stronger associations. These results warrant further evaluation in future studies.

## MATERIALS AND METHODS

### Subjects

This case-control study includes 1,028 incident UATC cases and 3,081 age- and sex-matched controls. Both cases and controls were selected from participants of the Hospital-based Epidemiologic Research Program at Aichi Cancer Center (HERPACC) between January 2001 and November 2005 at Aichi Cancer Center Hospital (ACCH) in Japan. Detail of HERPACC is described elsewhere [37]. Briefly, all first-visit outpatients (*n* = 29,736) during the study period were asked to complete self-administered questionnaire and provide blood samples. Of these, 28,776 (96.7%) agreed to participate and provided written informed consent. Of these, 14,329 subjects were not diagnosed as having cancer within the 1-year period before determining study eligibility. Among the remaining 14,447 subjects, 9,838 were diagnosed with incident cancer of any organ, 3,657 were prevalent cases and 952 were of undetermined status. Cases were selected from 9,838 incident cancer cases and controls from 14,329 participants who were determined not to have cancer.

Cases in this study were participants of HERPACC who were histologically diagnosed as having a newly incident UATC [cancer of head and neck in 595 (57.9%), cancer of esophagus in 433 (42.1%)] without any prior history of cancer. We defined UATC according to the following codes of the International Classification of Diseases and Related Health Problems (ICD10): cancers of the oral cavity and oropharynx (C00.3-C00.9, C01, C02.0-C02.4, C03, C04, C05.0-C05.2, C06, C09 and C10), hypopharynx (C12 and C13), oral cavity–oropharynx–hypopharynx if not otherwise specified (C02.8, C02.9, C05.8, C05.9 and C14), salivary glands (C07 and C08), nasopharynx (C11), larynx (C32) and esophagus (C15). Head and neck cancer was defined as UATC other than esophageal cancer. All subsites of UATC were frequency-matched on age and sex at a case-control ratio of 1:3.

Control subjects also were participants of HERPACC during the same period, but were confirmed to have no detectable cancer and no history of neoplasia. Non-cancer status was confirmed by medical examinations, including radiographic examinations when indicated. We applied individual matching for control selection on age (± 4 years)- and sex. They were selected randomly from non-cancer subjects among HERPACC participants in a case-control ratio of 1:3. The study was approved by the Institutional Ethics Committee at ACC.

### Data collection

Information on potential confounders, alcohol consumption, cumulative exposure to smoking, socioeconomic status (SES), number of teeth and flushing response after drinking of a glass of beer was collected using a self-administered questionnaire.

We grouped cumulative exposure to smoking status into four categories by pack-years (PY) as follows: nonsmoker, low-moderate smoker (PY<20), high-moderate smoker (20≤PY<40), and heavy smoker (PY ≥40). Daily alcohol consumption of various common beverages (Japanese *sake*, beer, *shochu*, whiskey and wine) was determined according to the average number of drinks per day, which was then converted into a Japanese *sake* (rice wine) equivalent measure of 180 ml; termed a *go*, which is a standard measure in Japan containing 23 g of ethanol. Drinking status in this study was classified into the four categories of never drinker, moderate drinker (less than 1 *go* on 5 days per week), high-moderate drinkers (1–2 *go* on 5 days per week) and heavy drinkers (more than 2 *go* on 5 days per week).

The intake of nutrients was measured using a semi-quantitative food frequency questionnaire (FFQ), described in detail elsewhere [38]. Briefly, the FFQ consisted of 47 single food items with eight frequency categories and three portion size categories (small, medium, and large) provided. We estimated average daily intake by multiplying the reported frequency of intake by the selected serving size of each food (g). Intakes of all food items were not-energy-adjusted. The FFQ was validated in a population similar to that of our study using a 3-day weighed dietary record as the reference standard; results indicated acceptable reproducibility and validity [39].

Participants also were asked about their occupation as a measure of SES and were categorized into three groups as follows: white collar, blue collar or others, including part-time employees, housewives, students, unemployed, retired and inactive. Number of teeth of participants were categorized into four groups as follows: 0, 1–8, 9–20, >21. Flushing response after drinking of a glass of beer was categorized as yes or no.

### Dietary inflammatory index

Dietary inflammatory index (DII^®^) scores were estimated based on self-reported macro- and micronutrients (energy, protein, fat, carbohydrate, total dietary fiber, cholesterol, saturated fatty acid, monounsaturated fatty acid, polyunsaturated fatty acid, n-3 polyunsaturated fatty acid, n-6 polyunsaturated fatty acid, carotene, vitamin B1, vitamin B2, vitamin B6, vitamin B12, vitamin C, vitamin D, vitamin E, retinoic acid, folate, and iron) based on the FFQ, described in detail elsewhere [38]. Briefly, dietary data were first linked to a regionally representative global database that provided a robust estimate of the mean and the standard deviation for each food parameter included in the DII. These parameters then became the multipliers to express an individual’s exposure relative to the “standard global mean” as a z-score. This was achieved by subtracting the “standard global mean” from the amount reported and dividing this value by the standard deviation. To minimize the effect of “right skewing,” this value was then converted to a centered (on zero) proportion by converting the z-score to a proportion (i.e., with values from 0 to 1) then multiplying by 2 and subtracting 1. The centered proportion score for each food parameter for each subject was then multiplied by the corresponding food parameter effect score in order to obtain a food parameter-specific DII score. All of the food parameter-specific DII scores were then summed to create the overall DII score for each subject. Alcohol was not included in the DII calculation, as it was adjusted separately in the analyses. The remaining 23 food parameters that were not used or were missing are mentioned in Supplementary Table 1.

### Statistical analyses

We evaluated the association between DII and UATC risk using quartiles of DII scores. Quartile thresholds for DII were based on the distribution of DII in matched controls. Odds ratios (ORs) and 95% confidence intervals (CI) were estimated using conditional logistic regression models, with the first DII quartile (Q1, most anti-inflammatory diet category) as the reference. In the logistic regression model, the linear association with DII was evaluated by including DII quartile as an ordinal score (1, 2, 3, and 4). Potential confounders considered in the adjusted analyses were smoking, alcohol consumption, flushing phenotype [40], number of teeth [41] and occupational group. We also examined the impact of DII according to anatomic subtypes of UATC. The impact of DII was evaluated with stratification by the above-mentioned confounders in UATC. Interactions were evaluated by the likelihood ratio test comparing a model including a cross-product term between variables of interest and DII quartiles and a model without the term.

Chi-square tests for categorical variables and ANOVA for continuous variables were used to assess difference across groups. Statistical analyses were performed using STATA^®^ SE version 13.1 (Stata Corporation, College Station, TX,

USA). *P*-Values less than 0.05 were considered significant.

## SUPPLEMENTARY MATERIALS TABLES


