# Supplementary material for: Dietary inflammatory index and risk of upper aerodigestive tract cancer in Japanese adults
Source: Oncotarget. 2018 May 8;9(35):24028–40. doi: 10.18632/oncotarget.25288 (PMC5963633; doi:10.18632/oncotarget.25288)
Supplement: Supplementary file 1 [file oncotarget-09-24028-s001.pdf]

Dietary inflammatory index and risk of upper aerodigestive tract cancer in Japanese adults

SUPPLEMENTARY MATERIALS

Supplementary Table 1: The remaining 23 food parameters that were not used or were missing

Food Parameter	Not available/used in the Japanese Head and Neck cancer study
1. Magnesium	Not available
2. Niacin	Not available
3. Alcohol	Not used
4. Selenium	Not available
5. Trans Fat	Not available
6. Zinc	Not available
7. Tea	Not available
8. Isoflavones	Not available
9. Pepper	Not available
10. ThymeOregano	Not available
11. Caffeine	Not available
12. Rosemary	Not available
13. Flavan3ol	Not available
14. Flavones	Not available
15. Flavonols	Not available
16. Flavonones	Not available
17. Anthocyanidins	Not available
18. Turmeric	Not available
19. Saffron	Not available
20. Onion	Not available
21. Garlic	Not available
22. Ginger	Not available
23. Eugenol	Not available

Supplementary Table 2: DII and estimated items among controls

	DII quartiles (median, interquartile range)				
**Estimated items**	**1**	**2**	**3**	**4**	***P* value**
Protein (g/d)	58.8, 13.8	53.9, 13.0	50.3, 11.6	46.0, 11.9	<0.001
Fat (g/d)	48.7, 14.	41.9, 11.7	39.0, 10.9	34.1, 10.6	<0.001
Carbohydrate (g/d)	238.4, 56.8	236.0, 61.4	231.3, 62.4	238.5, 76.2	0.011
Total dietary fiber (g/d)	13.5, 3.3	10.8, 2.6	9.2, 2.0	7.8, 2.2	<0.001
Cholesterol (mg/d)	267.8, 103.4	234.6, 99.4	227.2, 95.4	185.9, 77.5	<0.001
Saturated fatty acid (g/d)	12.0, 3.5	10.9, 3.3	10.3, 3.1	9.3, 3.1	<0.001
Monounsaturated fatty acid (g/d)	17.8, 5.4	15.1, 4.1	14.0, 12.5	12.5,2.7	<0.001
Polyunsaturated fatty acid (g/d)	15.1, 12.5	12.5, 3.5	11.5, 2.6	10.1, 2.5	<0.001
n-3 polyunsaturated fatty acid (mg/dl)	2694.3, 808.6	2279.1, 565.3	2083.5, 508.9	1732.6, 403.6	<0.001
n-6 polyunsaturated fatty acid (mg/d)	12305.8, 4095.8	10248.1, 3192.1	9489.0, 2650.1	8324.2, 2454.8	<0.001
Carotene (μg/d)	4302.7, 1456.7	2916.9, 961.4	2313.9, 776.9	1915.0, 374.8	<0.001
Vitamin B1 (mg/d)	0.7, 0.1	0.6, 0.1	0.6, 0.1	0.6, 0.1	<0.001
Vitamin B2(mg/d)	1.3, 0.3	1.1, 0.3	1.0, 0.3	0.9, 0.3	<0.001
Vitamin C (mg/d)	126.1, 43.5	97.5, 35.6	79.7, 31.8	60.8, 25.8	<0.001
Vitamin D (μg/d)	9.8, 4.7	8.2, 3.4	7.5, 3.2	4.8, 1.2	<0.001
Vitamin E (mg/d)	10.0, 2.5	8.0, 1.5	7.0, 1.4	6.0, 1.3	<0.001
Reti EQ (mg/d)	1270.9, 547.2	891.8, 468.4	690.9, 439.9	526.8, 174.4	<0.001
Folate (μg/d)	431.7, 104.3	335.3, 79.9	278.1, 69.6	222.8, 66.3	<0.001
Fe (μg/d)	8.8, 2.3	7.3, 1.8	6.4, 1.7	5.3, 1.5	<0.001
Meat (g/d)	35.5, 28.0	27.5, 23.5	26.5, 18.5	21.0, 13.5	<0.001
Fish (g/d)	50.0, 29.5	40.0, 23.5	36.5, 23.0	18.0, 6.5	<0.001
Seafoods other than fish (g/d)	19.5, 17.0	16.0, 11.0	14.0, 8.5	12.5, 7.5	<0.001
Green-yellow vegetables (g/d)	110.0, 55.0	64.0, 33.0	40.0, 23.0	27.0, 16.0	<0.001
Other vegetables (g/d)	89.5, 47.5	60.0, 34.5	43.5, 29.0	27.0, 20.5	<0.001
Fruits (g/d)	88.0, 78.0	58.0, 62.0	34.0, 47.0	25.0, 17.0	<0.001
Soy (g/d)	55.0, 36.0	44.5, 33.0	37.0, 32.0	31.0, 27.0	<0.001
